# Temperature and Relative Humidity Inside Trailers During Finishing Pig Loading and Transport in Cold and Mild Weather

**DOI:** 10.3390/ani4040583

**Published:** 2014-09-29

**Authors:** John McGlone, Anna Johnson, Avi Sapkota, Rebecca Kephart

**Affiliations:** 1 Animal and Food Sciences, Texas Tech University, Lubbock, TX 79409, USA; E-Mail: asapkota@purdue.edu; 2 Department of Animal Science, Iowa State University, Ames, IA 50011, USA; E-Mails: johnsona@iastate.edu (A.J.); rkdavis@iastate.edu (R.K.)

**Keywords:** bedding, internal trailer environment, loading, pig, relative humidity, transportation

## Abstract

**Simple Summary:**

The multi-site nature of the modern pork industry makes transport an essential part of swine production. It is well documented that transport induces stress in pigs. Bedding levels can have a significant effect on temperature and relative humidity inside the trailer. This study aims to determine the effects of bedding level on trailer temperature and humidity between average air temperatures of 4 °C and 18 °C. Relative humidity was greatest when higher levels of bedding were used during loading and transport in cold but not mild weather.

**Abstract:**

The effect of bedding levels and trailer compartment on internal trailer temperature and relative humidity (RH) during loading and transport of finishing pigs was evaluated in cold and mild weather. Three levels of bedding were used in each experiment: 0.6 m^3^, 1.2 m^3^, and 2.4 m^3^. In mild weather, internal temperatures were lower when 1.2 m^3^ or 2.4 m^3^ of bedding were used during loading and transport compared to 0.6 m^3^ (*P* < 0.05). Internal trailer temperature increased in a quadratic fashion in the top front compartment when 1.2 m^3^ was used (*P* < 0.05), and in a linear fashion in the top rear compartment when 2.4 m^3^ were used in cold weather (*P* < 0.05). In mild weather, temperature increased linearly in the top front compartment with heavy bedding levels. Relative humidity increased in a linear fashion in the top front compartment with 0.6 m^3^, bottom front with 1.2 m^3^, and top front with 1.2 m^3^ in cold weather (*P* < 0.05). In general, temperature and RH increased as bedding levels increased in both cold and mild temperatures. Excess bedding can absorb more moisture, resulting in transport loss and decreased animal welfare

## 1. Introduction

Temperature management is a crucial aspect of modern swine production. In today's production systems, most pigs are raised in indoor, climate-controlled buildings. The Ag Guide, published by Federation of Animal Science Societies (FASS), has recommended that pigs be raised in their thermal comfort zone [1]; however, pigs are transported for various purposes in all types of weather. While temperatures outside of the thermal comfort zone may induce stress in the pigs, transportation is an inevitable process in today’s swine production.

Transportation is stressful because of novelty, motion sickness, mixing of conspecifics, feed and water deprivation and sudden change in environment, to name a few reasons [2]. Wide variations in temperature and relative humidity (RH) are major factors affecting stress and welfare of pigs during transport. Based on the temperature heat index (THI), RH is more important in warm seasons [3,4].

The microenvironment inside the trailer is determined by temperature, RH and wind speed during transportation. The microenvironment is affected by stocking density, size of animals, ventilation, outside temperature and RH, and vehicle motion [5]. The level and quality of bedding used inside the trailer while transporting pigs is also a crucial factor because pigs are in direct contact with the bedding during transport.

Bedding material can hold moisture and thus affect the thermal comfort of the pigs depending on the season and outside air temperature [6]. Furthermore, trailers are divided into different compartments. These compartments may have different microenvironments depending on ventilation in that specific part of the trailer [7]. The objectives of this study are to determine the effect of bedding levels at different air temperatures and seasons in different compartments of a trailer while loading and transporting finishing pigs from finishing sites to the processing plant.

## 2. Experimental Section

### 2.1. General

The Institutional Animal Care and Use Committees at Texas Tech University and Iowa State University approved the experimental protocol for this study. This research was conducted using commercial finishing sites, trailers, and processing plants in the Midwest region of the U.S.

Because this is a large dataset with many variables, many more analyses are possible. The large dataset for this series of studies is available for others to analyze in different ways. Contact the corresponding author and the full dataset will be shared with qualified researchers or interested parties.

The study was conducted in January, February, March, and May, 2011. Pigs were transported in pot-bellied trailers [7,8] 14.7 m long and 2.6 m wide. Each trailer transported an average of 166.9 ± 0.63 pigs. Duration of travel varied due to varying distances between finishing sites and processing plants. Bedding was provided by the trucking company. Bedding levels were based on findings from McGlone *et al.* [9], and were randomly assigned to the trailers. Assigned levels were 0.6 m^3^ bedding, 1.2 m^3^ bedding, and 2.4 m^3^ bedding. All trailers had side-wall passive ventilation systems. Boarding levels for these vents were assigned following Transport Quality Assurance Handbook guidelines [10]. In Experiment 1 (<10 °C), side-wall vents were 50% open. In Experiment 2 (10 °C–25 °C), side-wall vents were 100% open.

### 2.2. Temperature and Relative Humidity

Sensors (Extech model RHT10, Extech Instruments and HOBO Model H08-003-02, Onset Computer Corp.) were installed on alternating sides in the front (5 m from front) and rear (5 m from rear) of each trailer at a height of 1 m above the floor of the top and bottom decks The sensors were set to record temperature and RH every 5 minutes from the time of start of loading until the time of end of unloading. One sensor was fastened on the outside of the trailer. The measurement zones were classified as bottom front (BF), bottom rear (BR), top front (TF), top rear (TR) and outside (OT). Time of start of loading, end of loading, arrival time at plant, waiting time at plant, start of unloading and end of unloading was recorded by the researchers.

### 2.3. Experiments

The study was divided into two experiments based on the average outside air temperature; Exp. 1: cold weather (less than 10 °C) and Exp. 2: mild weather (10 °C–25 °C). Transport was considered in two phases. Phase 1 was loading, which was defined as the time when the first pig placed its front foot inside the trailer to the time when the last pig placed its hind leg inside the trailer. Phase 2 was transport time, which was the time from when the trailer started moving from the finishing site to the time of arrival at the processing plant.

Because loading times varied, only the last 30 min of loading was considered for analyzing temperature and RH data in phase 1. For phase 2, temperature and RH were summarized starting 30 minutes after phase 1 and for the next 60 minutes thereafter. Any transport that was less than 120 minutes (most of which were less than 30 minutes) was not included in this study because the loading and temperature equilibration phase and wait time at the plant may overlap. A typical transport experience had a loading phase in which trailer temperature warms up. When the truck and trailer move, the trailer cools and reaches an equilibrium temperature (after 30 minutes) that depends primarily on outside temperature since trailers had a uniform number of pigs [9]. The trailer temperature then changes again when the truck and trailer stops and waits to unload and then it changes again during unloading.

### 2.4. Statistical Analysis

Data were transferred from sensors to Excel and analyzed using SAS 9.2 (SAS, 2010 SAS Inst., Inc., Cary, NC, USA). The model included bedding level, compartments in trailer, time, and their interactions as independent variables in each experimental dataset. Regression analyses were performed to determine whether temperature and RH varied over time in each compartment depending on each bedding level during loading. Linear and quadratic effects over time were calculated and separated using General Linear Model within SAS 9.2. The predicted difference test within SAS was used for multiple comparison corrections. Slope and SE of slopes (SEb) were determined using the REG procedure of SAS 9.2 (SAS, 2010 SAS Inst., Inc., Cary, NC, USA).

## 3. Results

Average temperature and RH for each bedding level, season (temperatures), and phase of the study is presented in Table 1. Variation in RH for each phase may be explained by the temperature and precipitation on the days that each phase was conducted.

**Table 1:** Environmental temperature and relative humidity by season outside of trailer during loading and transport.

Season/Temperature	Phase	N	Bedding level, (volume, m^3^)	Temperature [°C]	Relative Humidity [%]
Cold	Loading	7	0.6	4.30 ± 1.09	56.70 ± 6.27 ^a^
		14	1.2	4.20 ± 0.79	74.90 ± 4.56 ^b^
		12	2.4	4.10 ± 0.85	77.40 ± 5.80 ^b^
Cold	Transport	6	0.6	7.70 ± 1.74	55.80 ± 6.34
		12	1.2	4.40 ± 1.31	77.90 ± 4.79
		13	2.4	4.60 ± 1.10	75.10 ± 4.00
Mild	Loading	13	0.6	16.40 ± 0.71	49.60 ± 5.79
		9	1.2 or 2.4	14.00 ± 0.88	54.30 ± 6.61
Mild	Transport	10	0.6	18.00 ± 1.35	42.80 ± 7.07
		9	1.2 or 2.4	14.80 ± 1.48	59.00 ± 7.07

^a, b^ Least square means (temperature and relative humidity) within a column for each weather and event with different superscripts differ (*P* < 0.05). N = number of trailers.

**Table 2:** *P*-values of bedding, compartment, bedding by compartment (B × C), time, time by bedding (T × B), time by compartment (T × C) and time by bedding by compartment (T × B × C) effect on temperature (Temp) and relative humidity (RH) at each phase in each season/temperature.

				*P*-Values						
Season/Temperature	Phase	N	Measure	Bedding	Compartment	B×C	Time	T×B	T×C	T×B×C
Cold	Loading	33	Temp	0.02	0.07	0.33	<0.01	0.57	<0.01	0.99
			RH	<0.01	0.03	0.12	<0.01	<0.01	0.30	0.99
	Transport	31	Temp	0.13	0.83	1.00	-	-	-	-
			RH	0.06	0.98	0.99	-	-	-	-
Mild	Loading	22	Temp	<0.01	0.06	0.55	<0.01	<0.01	<0.01	0.45
			RH	0.20	0.06	0.97	<0.01	0.07	0.19	0.12
	Transport	19	Temp	<0.01	0.66	0.95	-	-	-	-
			RH	0.26	0.91	0.83	-	-	-	-

N = number of trailers.

The *P*-values of bedding level, compartment, bedding × compartment, time, and interactions of time × bedding level, time × compartment, and time × bedding × compartment effects during phase 1 and phase 2 in both experiments are presented in Table 2.

### 3.1. Cold Weather

During loading in cold weather, average internal trailer temperature was colder when 2.4 m^3^ of bedding were used (4.10 ± 0.60 °C) compared to when 1.2 m^3^ of bedding were used (6.30 ± 0.53 °C; *P* < 0.05). When 0.6 m^3^ of bedding were used, however, average internal trailer temperature (5.90 ± 0.90 °C) was not different from 1.2 m^3^ or 2.4 m^3^ (*P* > 0.05). Relative humidity inside the trailer was lower when 0.6 m^3^ of bedding were used compared to when 1.2 m^3^ and 2.4 m^3^ were used (56.10 ± 4.38, 75.00 ± 2.56, and 81.60 ± 2.93% for 0.6 m^3^, 1.2 m^3^, and 2.4 m^3^, respectively) (*P* < 0.05). Moisture percentage inside the trailer did not differ when 1.2 m^3^ and 2.4 m^3^ were used (*P* > 0.05).

During transport in cold weather, internal trailer temperature was greatest when 0.6 m^3^ of bedding were used (11.80 ± 0.76 °C) compared with when 1.2 m^3^ (6.20 ± 0.69 °C) or 2.4 m^3^ (5.90 ± 0.58 °C) were used. Temperatures when 1.2 m^3^ and 2.4 m^3^ were used did not differ from each other (*P* > 0.05). A similar pattern was found for RH (53.10 ± 2.68, 71.50 ± 2.44, and 73.10 ± 2.03% for 0.6 m^3^, 1.2 m^3^, and 2.4 m^3^, respectively) during transport in cold weather. Table 3 presents the pattern (linear, quadratic, or non-significant) in which temperature and RH change depending on bedding level and compartments in the trailer. *P* > 0.05 was considered non-significant.

**Table 3:** Linear (L), quadratic (Q) or non-significant (NS) change in temperature and relative humidity (RH) over time while loading in cold weather for 0.6 m^3^, 1.2 m^3^ and 2.4 m^3^ of bedding (n = 33 trailers).

	**Bedding volume (m^3^)**											
	0.6				1.2				2.4			
	BF^1^	BR^1^	TF^1^	TR^1^	BF^1^	BR^1^	TF^1^	TR^1^	BF^1^	BR^1^	TF^1^	TR^1^
**Temperature [°C]**	NS	NS	NS	NS	NS	NS	NS	Q	NS	NS	L	NS
**RH [%]**	NS	NS	L	NS	L	NS	L	NS	NS	NS	NS	NS

^1^ BF = bottom front, BR = bottom rear, TF = top front, TR = top rear compartments. Note: Significant Temperature and relative humidity over time (either linear or quadratic) have been plotted in Figure 7 and Figure 8.

### 3.2. Mild Weather

During loading in mild weather, internal trailer temperature was lower when 1.2 m^3^ or 2.4 m^3^ of bedding were used compared to when 0.6 m^3^ of bedding was used (12.90 ± 0.74 °C and 16.70 ± 0.47 °C, respectively; *P* < 0.05). A similar pattern was observed during transport in mild weather for 0.6 m^3^ and 1.2 m^3^ or 2.4 m^3^ of bedding level (17.90 ± 0.74 and 14.50 ± 0.74 °C, respectively; *P* < 0.05). Relative humidity did not differ with bedding level during loading and transport in mild weather.

Table 4 presents the pattern (linear, quadratic, or non-significant) in which temperature and RH change depending on bedding level and compartments in the trailer. *P* > 0.05 was considered non-significant.

**Table 4:** Linear (L), quadratic (Q) or non-significant (NS) change in temperature and relative humidity (RH) over time while loading in mild weather for 0.6 m^3^ and 1.2 m^3^ (n = 21 trailers).

	**Bedding volume (m^3^)**							
	0.6				1.2			
	BF^1^	BR^1^	TF^1^	TR^1^	BF^1^	BR^1^	TF^1^	TR^1^
**Temperature [°C]**	NS	NS	NS	NS	NS	NS	L	NS
**RH [%]**	NS	NS	NS	NS	NS	NS	Q	L

^1^ BF = bottom front, BR = bottom rear, TF = top front, TR = top rear compartments. Note: Significant Temperature and relative humidity over time (either linear or quadratic) have been plotted in Figure 9.

### 3.3. Variable Interactions

**Figure 1:**
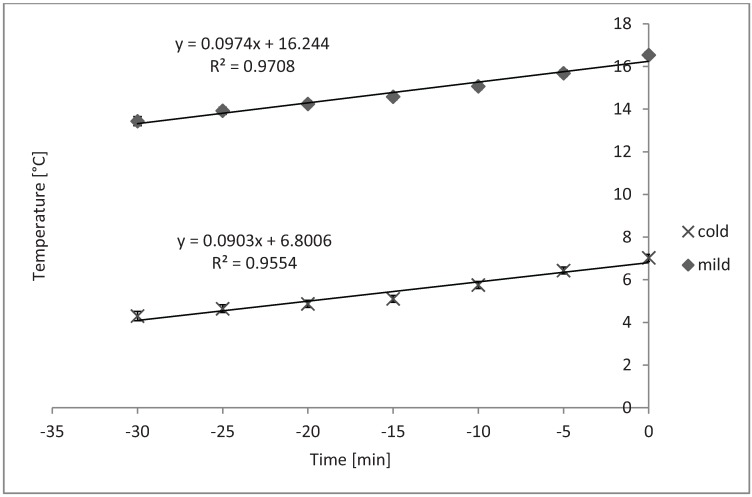
Overall linear time effect on average internal trailer temperature while loading during cold and mild weather (*P* < 0.01 and *P* < 0.01, respectively). Cold SEb = 0.06, n = 33 trailers; Mild SEb = 0.10, n = 21 trailers. Although slopes differed from zero, they did not differ between cold and mild seasons/temperatures (*P* = 0.56).

**Figure 2:**
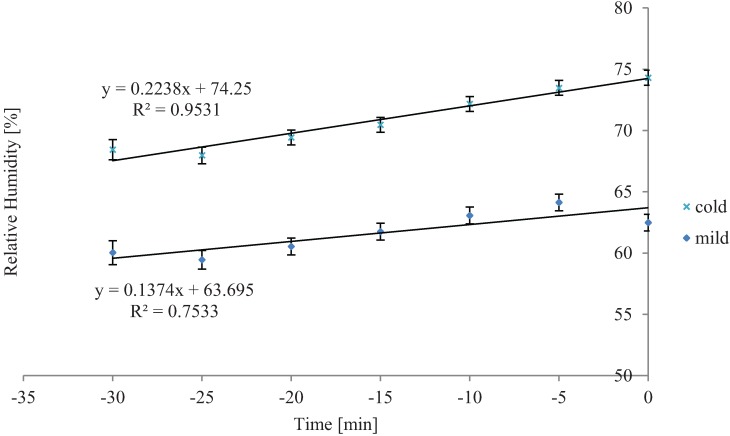
Overall time effect on average internal trailer relative humidity inside while loading during winter and mild weather (*P* < 0.01 and *P* < 0.01, respectively). Cold SEb = 0.02, n = 33 trailers; Mild SEb = 0.02, n = 21 trailers. While slopes differed from zero, they did not differ between cold and mild seasons/temperatures (*P* = 0.50). Time zero represents when the trailer departed and −30 minutes is the time loading began.

Average internal trailer temperature and RH increased linearly over time during loading in cold weather, and the slopes of these did not differ from each other (Figure 1 and Figure 2, respectively; *P* < 0.05). Trailers increase internal air temperature about 0.1 °C per minute in a linear fashion during loading (Figure 1). Likewise, trailer internal RH increased from 0.13% to 0.22% per minute during loading (Figure 2).

**Figure 3:**
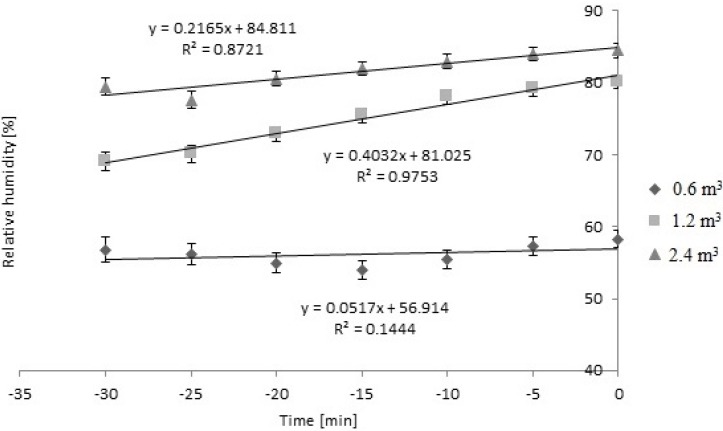
Bedding × time effect on relative humidity while loading during cold weather (*P* < 0.01). Three levels (0.6 m^3^, 1.2 m^3^, and 2.4 m^3^) of bedding were used. Slope was 0 when 0.6 m^3^ of bedding were used. Slopes differed from 0 when 1.2 m^3^, and 2.4 m^3^ of bedding were used (*P* < 0.05). 1.2 m^3^ SEb = 0.03, n = 14 trailers; 2.4 m^3^ SEb = 0.06, n = 12 trailers. Slopes of 1.2 m^3^ and 2.4 m^3^ did not differ from each other (*P* > 0.05).

As shown in Figure 3, there was a bedding × time effect on RH while loading in cold weather (*P* < 0.01).

Relative humidity inside the trailer did not change during loading in the winter season when 0.6 m^3^ of bedding were used (*P* > 0.05); however, RH increased linearly when 1.2 m^3^ and 2.4 m^3^ were used during loading (*P* < 0.05). The slopes did not differ from each other (*P* > 0.05).

**Figure 4:**
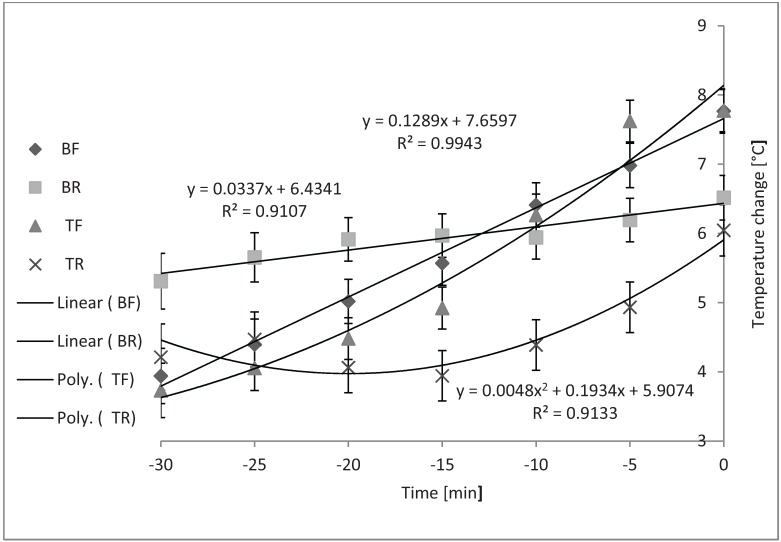
Compartment × time effect on average internal trailer temperature while loading during cold weather (*P* < 0.01; n = 33). Slopes of BF and BR were not different from zero. Slopes of TF (SEb < 0.01) and TR (SEb < 0.05), differed from zero, but were not different from each other.

Figure 4 shows that the compartment × time effect was also significant while loading in the winter season (*P* < 0.01). Slopes and correlations were found to be: y = 0.1289x + 7.6597, R^2^ = 0.9943 (BF); y = 0.0337x + 6.4341, R^2^ = 0.9107 (BR); and y = 0.0048x^2^ + 0.1934x + 5.9074, R^2^ = 0.9133 (TR).

BF = bottom front, BR = bottom rear, TF = top front, TR = top rear compartments.

Temperatures in the BF and BR compartments did not change over time. Temperatures in the TF and TR compartments changed in quadratic fashion, but the slopes did not differ from each other.

**Figure 5:**
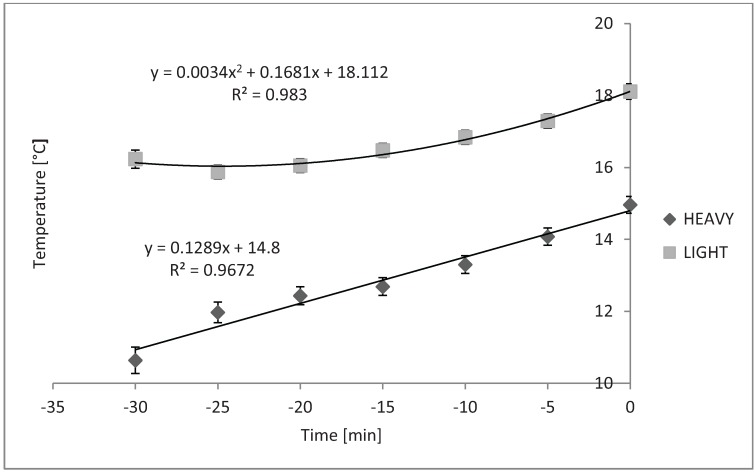
Time × bedding level effect on temperature while loading during mild weather (*P* < 0.01). 0.6 m^3^ (n = 13 trailers) and 1.2 m^3^ or 2.4 m^3^ (n = 9 trailers) bedding levels were used. 0.6 m^3^ SEb < 0.01 and 1.2 m^3^ or 2.4 m^3^ = 0.11. There was a quadratic change in internal trailer temperature when light bedding was used and linear increase in internal trailer temperature when heavy bedding was used during loading in mild season/temperatures (*P* = 0.04 and *P* < 0.01, respectively).

Figure 5 shows the time × bedding level effect on internal trailer temperature in mild weather (*P* < 0.01).

**Figure 6:**
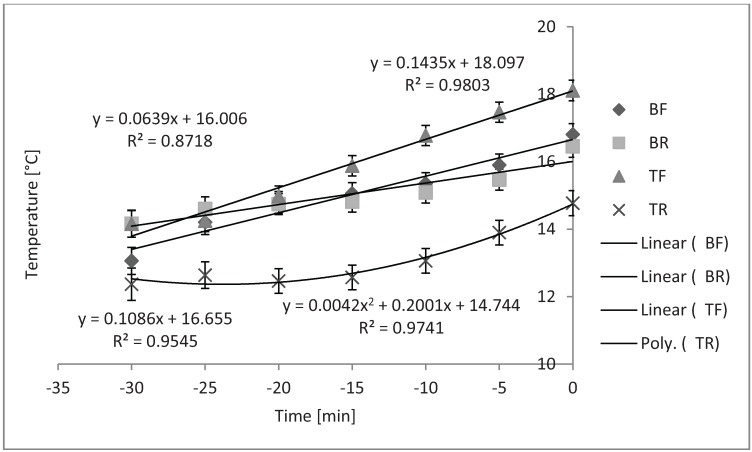
Compartment × time effect on internal trailer temperature while loading during mild weather (n = 21). Slopes of BF, BR and TR did not differ over time. Slope of TF was linear over time while loading in mild weather (*P* = 0.02, SEb = 0.14).

Internal trailer temperature changed in a quadratic fashion when 0.6 m^3^ were used and in a linear fashion when 1.2 m^3^ or 2.4 m^3^ were used in the trailers. The compartment × time effect was also significant during loading in mild weather, as shown in Figure 6 (*P* = 0.02).

BF = bottom front, BR = bottom rear, TF = top front, TR = top rear compartments.

**Figure 7:**
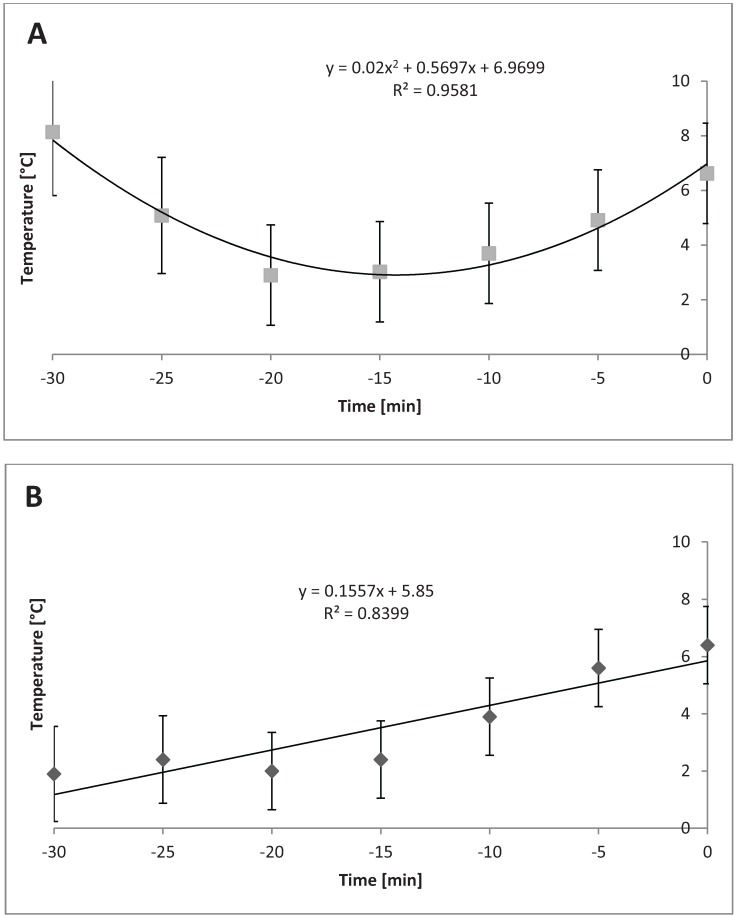
Temperature in cold weather in different compartments of trailer when different levels of bedding are used (n = 33 trailers). (**A**) Quadratic change in temperature in top rear compartment over time when using 1.2 m^3^ of bedding during cold (*P* = 0.02, SEb < 0.01). (**B**) Linear change in temperature in top front compartment when using 2.4 m^3^ of bedding during cold (*P* < 0.01, SEb = 0.18).

The regression slope for the BF, BR, and TR compartments did not differ from zero while slopes for the TF compartment was linear (*P* = 0.02). Figure 7A shows the quadratic change in temperature in the TR compartment during loading over time when 1.2 m^3^ of bedding were used in cold weather (*P* = 0.02). Temperature in the TF compartment changed linearly over time when 2.4 m^3^ of bedding were used, as shown in Figure 7B (*P* < 0.01).

**Figure 8:**
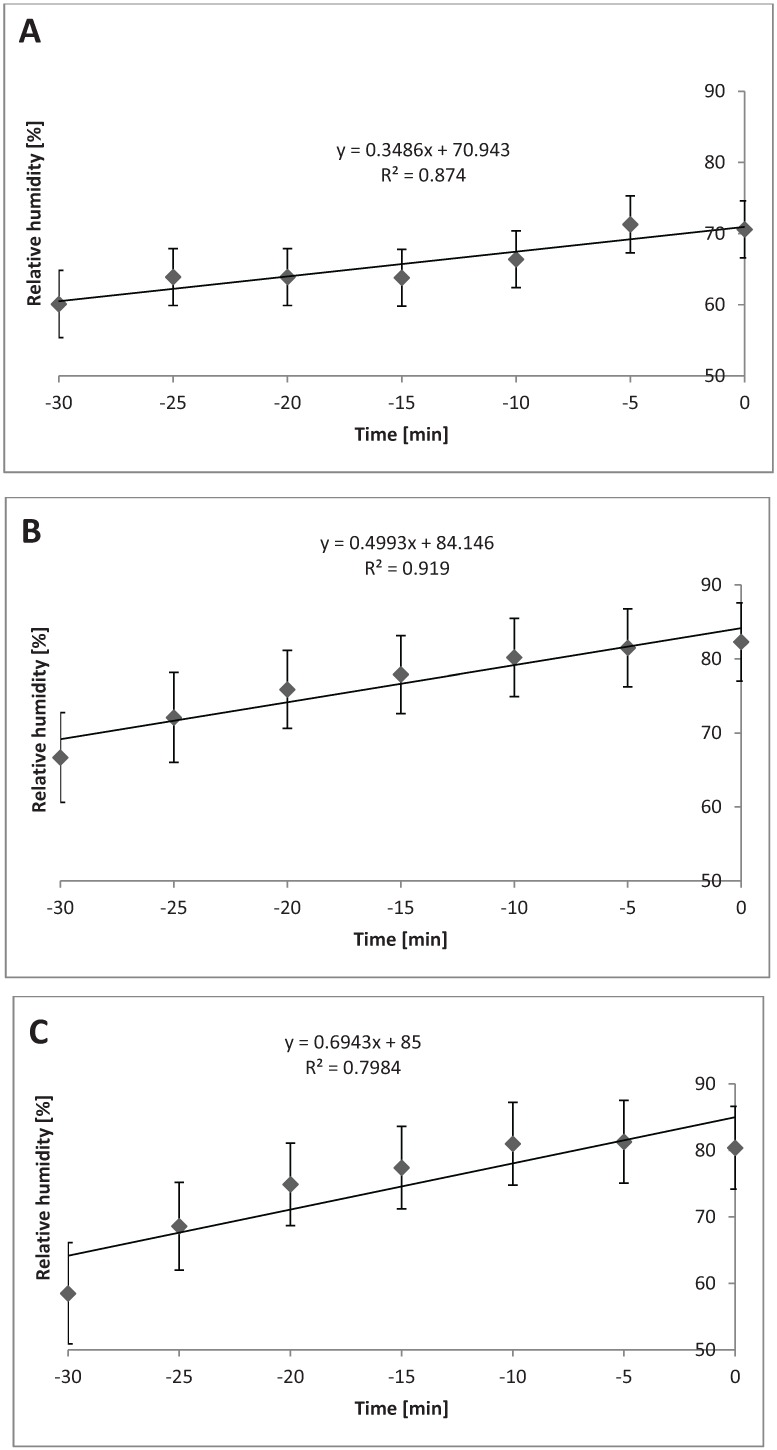
Relative humidity in cold weather (n = 33 trailers) in different compartments of trailer when different levels of bedding are used. (**A**) Linear change in relative humidity in top front compartment while using 0.6 m^3^ bedding during cold (*P* = 0.03, SEb = 0.54). (**B**) Linear change in relative humidity in bottom front compartment when using 1.2 m^3^ bedding during cold (*P* = 0.02, SEb = 0.70). (**C**) Linear change in relative humidity in top front compartment when using 1.2 m^3^ bedding during cold (*P* = 0.01, SEb = 0.84).

Relative humidity in the compartments at different bedding levels in cold season/temperatures changed linearly over time during loading in the following scenarios: TF with 0.6 m^3^ of bedding (*P* = 0.03); BF with 1.2 m^3^ (*P* = 0.02); and TF with 1.2 m^3^ (*P* = 0.01). Figures 8A, 8B, and 8C show each of these relationships.

**Figure 9:**
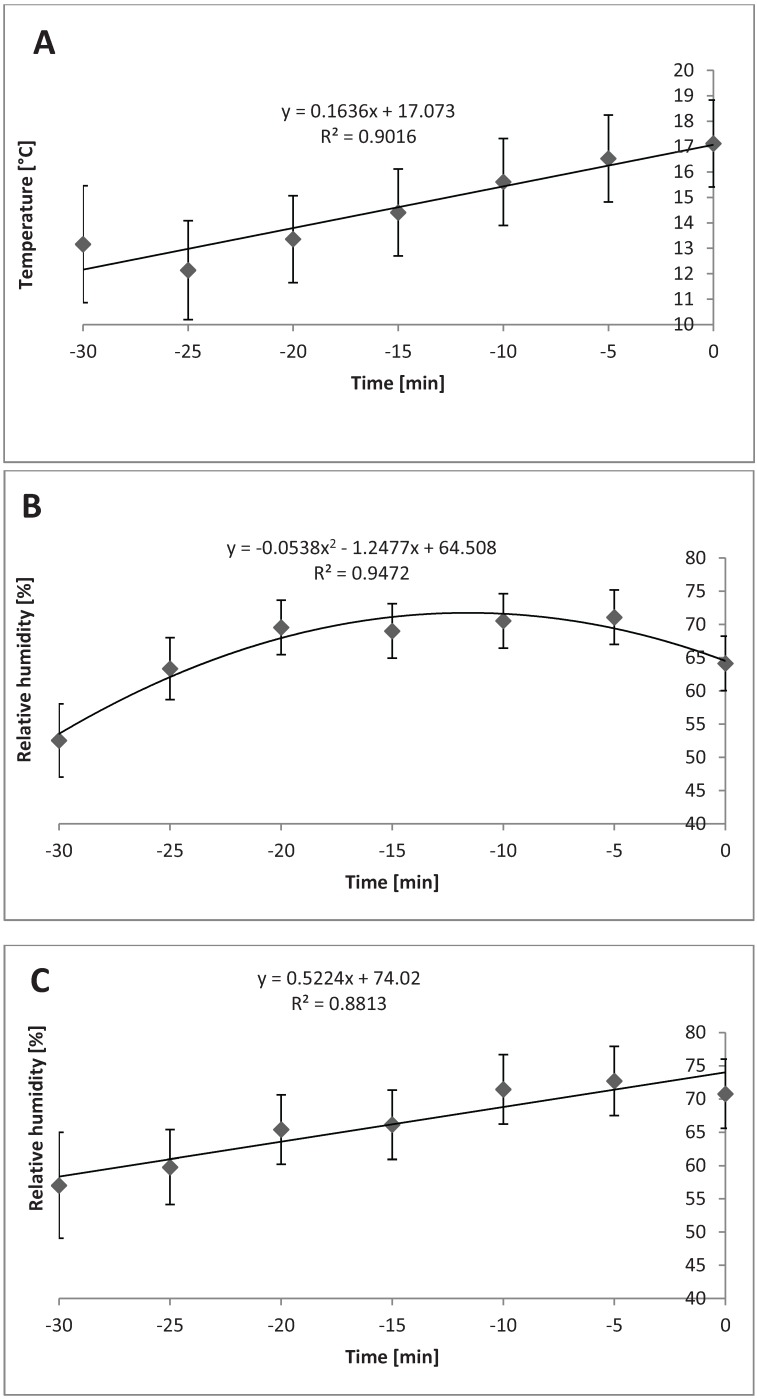
Temperature and relative humidity in mild weather (n = 21 trailers) in different compartments when different levels of bedding are used. (**A**) Linear change in temperature in top front compartment when 1.2 m^3^ or 2.4 m^3^ are used during mild weather (*P* = 0.02; SEb = 0.23). (**B**) Quadratic change in relative humidity in top front compartment when 1.2 m^3^ or 2.4 m^3^ are used during mild weather (*P* < 0.01; SEb = 0.02). (**C**) Linear change in relative humidity in top rear compartment when 1.2 m^3^ or 2.4 m^3^ are used during mild weather (*P* = 0.03; SEb = 0.72).

In mild weather, temperature in the TF compartment increased linearly over time when 1.2 m^3^ or 2.4 m^3^ were used (*P* = 0.02), as shown in Figure 9A. Figure 9B shows the quadratic change in relative humidity in the TF compartment when heavy bedding was used (*P* < 0.01). Relative humidity changed linearly in the TR compartment when 1.2 m^3^ or 2.4 m^3^ were used, shown in Figure 9C (*P* = 0.03).

## 4. Discussion

Transportation is already established as a stressor in pigs, but is a multi-factorial problem. Because pigs lack effective anatomical mechanisms (that is, functional sweat glands) to conserve or lose heat when necessary, air temperature and RH outside and inside the trailer in which pigs are transported are important variables influencing transportation loss as well as pig welfare. Current TQA guidelines suggest using different levels of bedding depending on the outside air temperature [10]. Amount and quality of bedding used can also affect internal trailer temperature and RH.

One factor affecting internal trailer temperature and RH is trailer design. Different trailer designs are used to transport pigs from finishing sites to commercial processing plants. Straight deck and pot-bellied trailers are the most commonly used types in the U. S. This study was conducted with pot-bellied trailers in a commercial setting. The trailer may be divided into several compartments based on design. Internal trailer temperature can vary with the stocking density, size of the pigs, ventilation and boarding, and outside air temperature.

In the present study, it was found that bedding level affected the average internal trailer temperature while loading both during cold and mild weather, and RH was affected by bedding level while loading during cold weather. Relative humidity was greatest when higher levels of bedding were used during loading and transport in cold weather but did not differ with bedding level in mild weather. When the temperature is cold, high humidity can have a greater chilling effect on pigs, because high levels of bedding can absorb more moisture. Because pigs are in direct contact with bedding containing high levels of moisture, they are more susceptible to frost bite, leading to welfare issues as well as increasing transport losses.

Compartment effect was significant on RH only during loading in cold weather which might be a result of compartment location and difference in boarding for each compartment. There was no specific order of compartments in which pigs were loaded. This varied with drivers and this might have masked the effect of compartment on temperature in each location. In addition, the sensors were installed inside the side walls of the trailer. Developing a better technique to give a better picture of each compartment could overcome this problem. In addition, if more sensors could be used to represent more locations inside the trailer, effect of location (compartment) would be more obvious. Hayne [11] installed sensors in 10 locations inside a trailer and reported that temperatures inside the trailer varied in different seasons/temperatures.

Most temperature and RH changes that are dependent on bedding level and compartment were not significant because temperature and RH are more interrelated at higher temperatures than when air temperature is lower based on Temperature Heat Index (THI). Effect of bedding level and compartments would be more obvious in warm seasons (temperature higher than 25 °C). Pigs are loaded and transported in the trailers such that they cannot lie down, and because they lack active sweat glands, maintaining proper internal trailer temperature and RH is more crucial in summer. Lewis [12] reported that internal trailer temperature increased linearly from 23.5 °C to 25.0 °C when a fully loaded trailer was stopped for 16 minutes, and noted a mean rise of 1.5 °C every 15 min when the trailer was stationary. Haley [13] reported that the transportation loss increased by three-fold when internal trailer temperature increased from a range of 8.6 °C–23.3 °C to a range of 23.4 °C–26.1 °C suggesting that an increase in temperature results in greater economic loss. Designing the trailers such that heat generated inside the trailer can be dissipated outside in an effective manner during summer could decrease transportation losses.

In the current study, in a commercial setting, relative humidity inside the trailer was greater when higher levels of bedding were used, implying poor animal welfare in cold weather. This suggests that a higher bedding level can aggravate the condition in warm weather, considering that higher bedding levels conserve more heat, and that pigs have a poor mechanism for evaporative loss of water to cope with the higher temperature. This study should be replicated in different climates to provide comprehensive information throughout the world.

## 5. Conclusions

There were changes in temperature in the top front compartment (quadratic change) and the top rear compartment (linear change) when higher amounts of bedding were used in cold weather. Relative humidity increased linearly in the top front compartment when a lower bedding level was used and the bottom front and top front compartments when medium bedding levels were used. In mild weather, temperature changed linearly in the top front compartment when heavy bedding was used. Designing the study using more sensors and in a controlled manner rather than in a commercial setting would help us better understand the effect of temperature and bedding on pig welfare.
